# Dynamic changes in lymphocyte subsets and parallel cytokine levels in patients with severe and critical COVID-19

**DOI:** 10.1186/s12879-021-05792-7

**Published:** 2021-01-18

**Authors:** Yangli Liu, Weiping Tan, Haihong Chen, Ying Zhu, Li Wan, Ke Jiang, Yubiao Guo, Kejing Tang, Canmao Xie, Hui Yi, Yukun Kuang, Yifeng Luo

**Affiliations:** 1 Division of Pulmonary and Critical Care Medicine, The First Affiliated Hospital of Sun Yat-sen University, Institute of Pulmonary Diseases, Sun Yat-sen University, Guangzhou, 510080 Province Guangdong People’s Republic of China; 2 Department of Radiology, The First Affiliated Hospital of Sun Yat-sen University, Guangzhou, 510080 Province Guangdong People’s Republic of China; 3 Thoracic surgery department, Union Hospital, Tongji Medical College, Huazhong University of Science and Technology, Wuhan, 430000 Province Hubei People’s Republic of China

**Keywords:** COVID-19, SARS-CoV-2, Dynamic changes, Lymphocyte subsets, Cytokine storm

## Abstract

**Background:**

The lack of knowledge regarding the pathogenesis and host immune response during SARS-CoV-2 infection has limited the development of effective treatments. Thus, we longitudinally investigated the dynamic changes in peripheral blood lymphocyte subsets and parallel changes in cytokine levels in COVID-19 patients with different disease severities to further address disease pathogenesis.

**Methods:**

A total of 67 patients (10 moderate, 38 severe and 19 critical cases) with COVID-19 admitted to a tertiary care hospital in Wuhan from February 8th to April 6th, 2020 were retrospectively studied. Dynamic data of lymphocyte subsets and inflammatory cytokines were collected.

**Results:**

On admission, compared with moderate cases, severe and critical cases showed significantly decreased levels of total lymphocytes, T lymphocytes, CD4^+^ T cells, CD8^+^ T cells, B cells and NK cells. IL-6 and IL-10 were significantly higher in the critical group. During the following hospitalization period, most of the lymphocyte subsets in the critical group began to recover to levels comparable to those in the severe group from the fourth week after illness onset, except for NK cells, which recovered after the sixth week. A sustained decrease in the lymphocyte subsets and an increase in IL-6 and IL-10 were observed in the nonsurvivors until death. There was a strong negative correlation between IL-6 and IL-10 and total lymphocytes, T lymphocytes, CD4^+^ T cells, CD8^+^ T cells and NK cells.

**Conclusions:**

A sustained decrease in lymphocyte subsets, especially CD4^+^ T cells and NK cells, interacting with proinflammatory cytokine storms was associated with severe disease and poor prognosis in COVID-19.

## Background

The disease caused by severe acute respiratory syndrome coronavirus 2 (SARS-CoV-2), named coronavirus disease 2019 (COVID-19), still poses a significant threat to public health worldwide. Prevention of acute respiratory distress syndrome (ARDS) and death in COVID-19 patients is an urgent health emergency. Proper treatment requires a better understanding of the pathogenesis of the disease. However, while the clinical features of COVID-19 and the biological characteristics of the virus are well documented [1–4], the pathogenesis and host immune response during SARS-CoV-2 infection have been poorly studied.

A rapid reduction in peripheral blood lymphocytes, especially T lymphocyte subsets, was observed and confirmed to predict pneumonia development and progression to respiratory failure at the early phase of the disease (within 2 weeks after onset) caused by severe acute respiratory syndrome coronavirus in 2003 [5] and Middle East respiratory syndrome (MERS-CoV) [6]. Recent studies have also reported decreased lymphocytes, particularly CD4^+^ and CD8^+^ T cells, in the peripheral blood of COVID-19 patients were in correlation with disease severity [7–10].

Cytokine storms are also considered to be one of the major causes of ARDS or extrapulmonary multiple-organ failure, and the degree of serum cytokine levels is positively correlated with mortality rate [11, 12]. Many critically ill COVID-19 patients who suddenly deteriorated in the later stages of the disease or during recovery were found to have cytokine storms [13]. High serum levels of inflammatory mediators and severe lymphopenia suggested an unbalanced deleterious immune response in COVID-19 [1]. A study involving three COVID-19 cases found that the early immune response in COVID-19 patients was highly dynamic. Parallel changes in cytokines and lymphocyte subsets suggested that the proinflammatory response may be intertwined with T lymphocyte activation, possibly exacerbating the disease and prolonging infection duration [14].

However, information is lacking regarding how T cell responses synchronize with cytokine levels and their interactions at the acute and convalescent phases of SARS-CoV-2 infection. In this study, we longitudinally investigated the dynamic changes in peripheral blood lymphocyte subsets and parallel changes in cytokine levels in COVID-19 patients with different disease severities to further address disease pathogenesis.

## Methods

### Study population

This study was a single-centre retrospective study and was approved by the Ethical Committee of Human Experimentation in the Union Hospital, Tongji Medical College, Huazhong University of Science and Technology. From February 8th to April 6th, 2020, a total of 85 hospitalized patients were laboratory diagnosed with COVID-19 after examination of SARS-CoV-2 by reverse transcription-polymerase chain reaction (RT-PCR) test from pharyngeal swab specimens in the isolation ward of the neurosurgery and thoracic surgery department of Union Hospital, Tongji Medical College, Huazhong University of Science and Technology. The diagnosis of COVID-19 was made according to the guidelines released by National Health Commission & State Administration of Traditional Chinese Medicine (Version 7, 15]. Of them, 18 patients were excluded due to a lack of complete results for lymphocyte subset levels and inflammatory cytokines. Sixty-seven patients were included in this study and were retrospectively reviewed.

### Data collection

Data from the electronic medical records system were extracted and analysed by two independent researchers. Information was collected on age, sex, days before hospitalization, hospitalization length of stay, comorbidities (including hypertension, diabetes mellitus, malignancy, chronic kidney disease and chronic lung disease), treatment and outcomes. All patients underwent routine laboratory tests, including complete blood count, liver and renal function and coagulation profile. Total lymphocytes, lymphocyte subsets (T lymphocytes, CD4^+^ T cells, CD8^+^ T cells, B cells and NK cells) and inflammatory cytokines (IL-2, IL-4, IL-6, IL-10, TNF-α and IFN-γ) were first detected on the day of admission before treatment and then once a week on average during hospitalization.

### Lymphocyte subset analysis

Lymphocyte subset levels were measured by BD FACS Canto flow cytometer (BD Biosciences, USA) according to the manufacturer’s instructions. In brief, 50 μl of whole blood was added to a BD Trucount tube and labelled with BD Multitest 6-colour (CD3/CD16 + 56/CD45/CD4/CD19/CD8) TBNK reagent (BD Biosciences, USA) for 15 min in darkness at room temperature. Then, after adding 450 μl of lysing solution, the samples were analysed with BD FACSCanto clinical software. The normal reference ranges of total lymphocytes, T lymphocytes (CD3^+^), CD4^+^ T cells (CD3^+^CD4^+^), CD8^+^ T cells (CD3^+^CD8^+^), B cells (CD3^−^CD19^+^) and NK cells (CD3^−^CD16^+^CD56^+^) were 1000–3300, 603–2993, 441–2156, 125–1312, 107–698 and 95–640 per μL, respectively.

### Cytokine detection

The concentrations of plasma cytokines (IL-2, IL-4, IL-6, IL-10, TNF-α and IFN-γ) were detected with a BD CBA human Th1/Th2 cytokine kit by a BD FACS Canto flow cytometer (BD Biosciences, USA) according to the manufacturer’s instructions. Briefly, 3 ml of venous blood was collected, and sera were obtained by blood centrifugation at 3500 r/min for 15 min. Six bead populations with significant fluorescence intensity (FL4) were coated with the abovementioned cytokine-specific PE-conjugated antibodies. Next, 50 μl of the cytokine capture beads were mixed with 50 μl of human Th1/Th2-II PE Detection Reagent and were then incubated with recombinant standards or samples to form sandwich complexes. Subsequently, the assay tubes were incubated for 3 h at room temperature on a non-absorbent, dry surface. Then, 1 ml of wash buffer was added to each assay tube and centrifuged at 200 g for 5 min. After adding 300 μl of wash buffer, the assay tubes were detected on a flow cytometer using FCAP Array software (Soft Flow Inc., Pecs, Hungary) to generate results in graphical and tabular format. The limits of detection for all cytokines were 1–5000 pg/ml. The normal reference ranges of IL-2, IL-4, IL-6, IL-10, TNF-α and IFN-γ were 0–4.1, 0–3.2, 0–2.9, 0–5.0, 0–23.0 and 0–18.0 (pg/ml), respectively.

### Definitions

The patients were divided into three groups according to the guidelines [15]: mild group, mild clinical symptoms with no abnormal radiological findings; moderate group, fever and other respiratory tract symptoms with pneumonia on chest computed tomography; severe group, meeting any of the following: (1) respiratory distress, respiratory rate ≥ 30/min; (2) oxygen saturation on room air ≤93% at rest; (3) PaO_2_/FIO_2_ ≤ 300 mmHg; or (4) lung infiltrates > 50% within 24 to 48 h; and critical group, meeting any of the following: (1) respiratory failure requiring mechanical ventilation; (2) shock; or (3) other organ failure requiring monitoring and treatment in the intensive care unit (ICU).

### Statistical analysis

Statistical analyses were performed with SPSS 22.0 (SPSS, Chicago, IL). Nonparametric variables are described as medians and quartile intervals (IQRs) and were compared using the Mann-Whitney U or Kruskal-Wallis test. Categorical variables are expressed as numbers and percentages [n (%)] and were compared using contingency table analysis and χ2 tests. The level of IL-6 was analysed after log10 transformation. A *p*-value of less than 0.05 was considered statistically significant.

## Results

### Baseline characteristics of COVID-19 patients

Sixty-seven patients hospitalized with COVID-19 diseases were included, with 10 in the moderate group, 38 in the severe group, and 19 in the critical group. The median age was significantly higher in the severe and critical groups than in the moderate group (65.0 y, 66.0 y vs. 39.5 y, *p* = 0.001). There was no significant difference in sex, days from illness onset to admission, or comorbidity distribution between the three groups. Hospitalization length of stay was much longer in the severe and critical group than in the moderate group (37.0 (IQR, 19.8–46.3), 44.0 (IQR, 27.0–50.0) vs. 16.5 (IQR, 10.0–20.3), *p* = 0.003). Most patients received empirical broad-spectrum antibiotics (moxifloxacin and/or cephalosporin) with a combination of antiviral treatment (arbidol) at the early phase (within 2 weeks after onset) of the disease. Sixteen (84.2%) of the critical patients, 20 (52.6%) of the severe patients and 1(10.0%) of the moderate patients received empirical treatment with prednisolone 0.5 mg/kg/day for no longer than 7 days after admission. Four patients (21.1%) in the critical group died in the hospital (Table 1).

**Table 1:** Comparison of baseline characteristics, immunological and cytokine parameters between patients with COVID-19

Variables	Moderate (*n* = 10)	Severe (*n* = 38)	Critical (*n* = 19)	*P* value
Age, y	39.5 (36.0–62.5)	65.0 (57.0–72.3)*	66 (56.0–70.0)*	0.001
Male	6	19	14	0.230
Days from illness onset to admission, d	22.5 (14.0–32.0)	14.0 (10.0–23.0)	14.0 (10.0–20.0)	0.153
Length of stay, d	16.5 (10.0–20.3)	37.0 (19.8–46.3) *	44.0 (27.0–50.0) *	0.003
Comorbidities				
Hypertension	4 (40.0)	16 (42.1)	12 (63.2)	0.282
Diabetes mellitus	1 (10.0)	12 (31.5)	3 (15.8)	0.225
Malignancy	1 (10.0)	2 (5.3)	0 (0.0)	0.436
Chronic kidney disease	0 (0.0)	2 (5.3)	1 (5.3)	0.759
Chronic lung disease	0 (0.0)	5 (13.2)	5 (26.3)	0.150
Treatment				
Antibiotics	9 (90.0)	28 (73.7)	18 (94.7)	0.115
Antiviral treatment	7 (70.0)	34 (89.5)	18 (94.7)	0.137
Corticosteroid	1 (10.0)	20 (52.6)	16 (84.2)	0.001
Death	0 (0.0)	0 (0.0)	4 (21.1)	0.01
Cell counts, per μL				
Total lymphocytes	2005 (1635–2458)	1280 (950–1550) *	800 (510–1050) # *	< 0.001
T lymphocytes	1413 (1236–2010)	821 (609–1142) *	639 (443–725) # *	0.002
CD4^+^ T-cell	940 (664–1292)	522 (358–735) *	377 (302–467) # *	0.001
CD8^+^ T-cell	443 (317–647)	243 (183–394) *	213 (78–264) *	0.002
B cell	221 (135–377)	137 (84–210) *	97 (45–128) *	0.001
NK-cell	137 (96–208)	118 (77–189)	39 (19–65) # *	0.001
Inflammatory cytokine				
C-reactive protein, mg/L	3.9 (0.1–15.5)	10.5 (4.2–25.7)	40.0 (15.9–58.7)# *	0.004
IL-2 (pg/ml)	3.6 (3.0–4.2)	2.9 (2.1–4.0)	3.0 (2.1–3.8)	0.339
IL-4 (pg/ml)	2.9 (1.9–3.9)	3.0 (1.3–3.8)	2.5 (1.4–3.9)	0.844
IL-6 (pg/ml)	5.8 (3.4–8.0)	6.8 (4.3–16.2)	14.5 (5.7–52.3) *	0.043
IL-10 (pg/ml)	4.0 (3.1–5.1)	4.5 (3.0–5.9)	5.4 (3.4–7.5) *	0.049
TNF-α (pg/ml)	2.7 (1.7–3.4)	2.1 (1.6–3.2)	2.6 (1.7–3.2)	0.325
IFN-γ (pg/ml)	2.6 (2.3–3.7)	3.1 (1.4–4.1)	2.8 (1.5–3.8)	0.527

Values are median (IQR) or n (%). * and # refer to *P* < 0.05. *Comparison between the moderate group and the severe or critical group. # Comparison between the severe group and the critical group

*IL* Interleukin; *TNF-α* Tumor necrosis factor α; *IFN-****γ*** Interferon **γ**

### Lymphocyte subset levels and inflammatory status on admission

Absolute counts of lymphocyte subsets were compared in the three groups on admission before treatment (Table 1). The median absolute total lymphocytes, T lymphocytes and CD4^+^ T-cells were significantly lower in severe cases (1280, 821,522, per μL, respectively) than in moderate cases (2005, 1413 and 940 per μL, respectively). They were reduced more profoundly in critical cases (800, 639 and 377, per μL, respectively). CD8^+^ T cells and B cells were significantly decreased in critical cases compared with moderate cases (213 and 97 per μL vs. 443 and 221 per μL, respectively, *P* < 0.01), while no significant difference was found between the severe and critical groups. NK cells were significantly reduced in the critical group compared to both the moderate and severe groups (39 vs. 137,118, *P* = 0.001). No significant difference was observed in the percentages of T lymphocytes, CD4^+^ T-cells, CD8^+^ T-cells, CD4^+^/CD8^+^ ratio, B cells or NK cells between the three groups (*P* > 0.05, data not shown).

Plasma cytokine levels on admission were further examined (Table 1). C-reactive protein (CRP) was significantly elevated in critical cases compared to moderate and severe cases (*p* = 0.004). The levels of interleukin 6 (IL-6) and IL-10 were markedly higher in critical cases than in moderate cases (14.5 and 5.4 pg/ml vs. 5.8 and 4.0 pg/ml, *p* < 0.05). No difference was found between the three groups in the levels of IL-2, IL-4, tumour necrosis factor α (TNF-α) and interferon γ (IFN-γ) on admission.

### Dynamic changes in lymphocyte subsets in severe and critical COVID-19

**Fig. 1:**
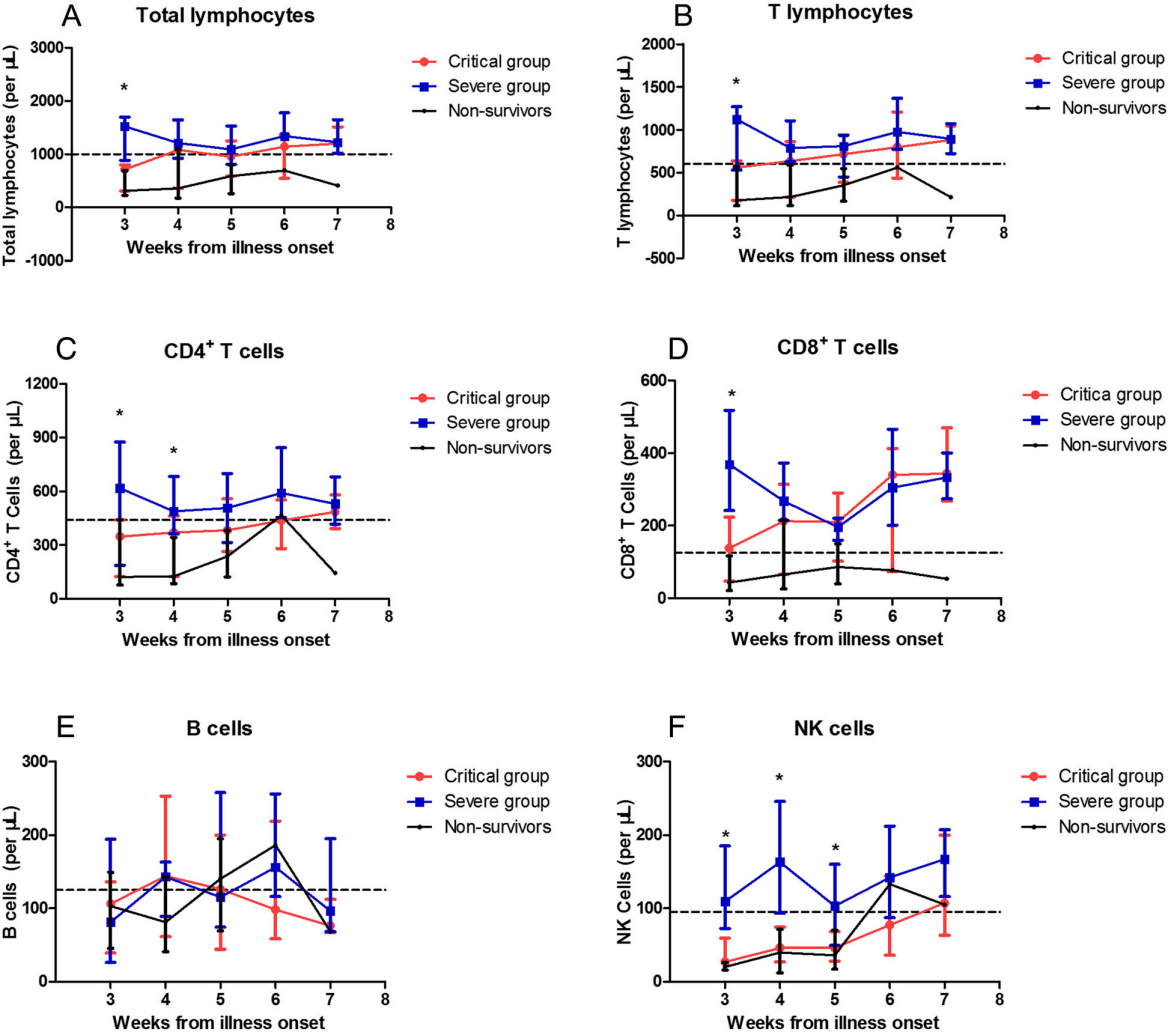
Dynamic changes of lymphocyte subsets in severe and critical COVID-19. Absolute numbers of total lymphocytes (**a**), T lymphocytes (**b**), CD4^+^ T-cells (**c**) and CD8^+^ T-cells (**d**), B cells (**e**), NK cells (**f**) were analyzed at different time points after hospital admission. Data were reported as median and interquartile range (IQR). The dotted line shows the lower limit of normal value of each parameter. *:*p* < 0.05

The moderate group was not included in this part because dynamic data were not available. Since the median time from illness onset to admission was 14 days in the severe and critical groups, the lymphocyte subset data in the first 2 weeks were too limited to analyse. We observed the dynamic changes in SARS-CoV-2 infection for the severe and critical groups starting from week 3. Additionally, data from four nonsurvivors are presented (Fig. 1).

Significant decrease in total lymphocytes, T lymphocytes and CD8^+^ T cells were observed in the critical group in the first 3 weeks. Then, they gradually increased and reached levels comparable to those in the severe group from the fourth week after illness onset, after which the median returned to the lower limit of normal (LLN) value (Fig. 1a, b, d). The decrease in CD4^+^ T cells lasted longer in the critical group for over 4 weeks and did not return to LLN until week 6 (Fig. 1c). However, a sustained decrease in the total lymphocyte, T lymphocyte, CD4^+^, and CD8^+^ T cell groups was observed in the nonsurvivors until death. A significant reduction in NK cells lasted even longer until week 5 for the critical group, and they began to exceed LLN from week 6. A similar trend was observed in the nonsurvivors (Fig. 1f). B cells fluctuated near the LLN, and no significant differences were found between the two groups over the whole course of the disease (Fig. 1e).

### Dynamic changes in inflammatory cytokines in severe and critical COVID-19

**Fig. 2:**
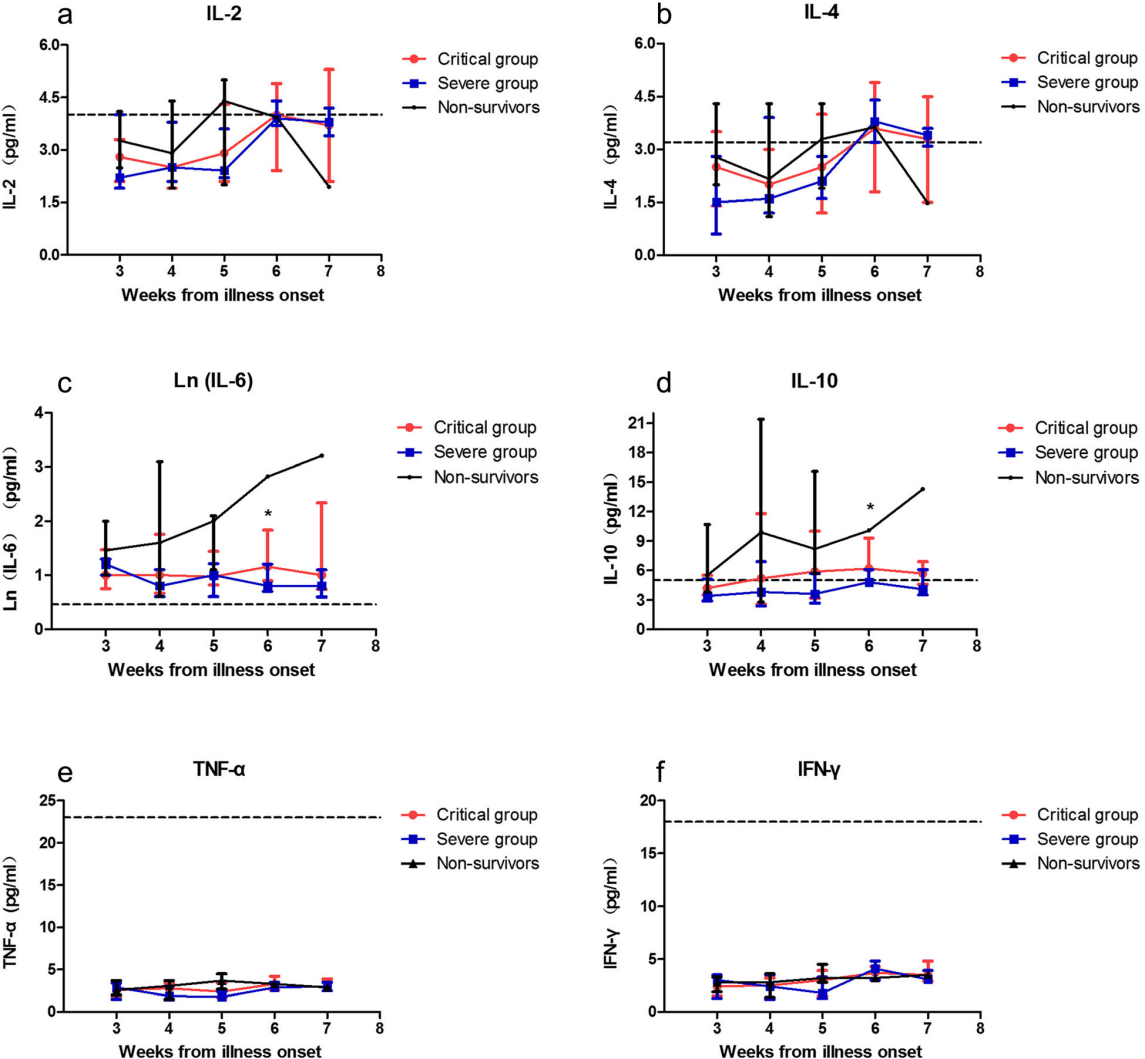
Dynamic changes of inflammatory cytokines in severe and critical COVID-19. Serum levels of IL-2 (**a**), IL-4 (**b**), Ln(IL-6) (**c**), IL-10 (**d**), TNF-α(**e**) and IFN-γ (**f**) were analyzed at different time points after hospital admission. Data were reported as median and interquartile range (IQR). The dotted line shows the upper limit of normal value of each parameter. *:*p* < 0.05

We further analysed the parallel changes in serum inflammatory cytokine levels, including IL-2, IL-4, IL-6, IL-10, IFN-γ and TNF-α (Fig. 2). No significant difference was observed between the critical and severe groups in the dynamic changes in IL-2, IL-4, TNF-α and IFN-γ. IL-2 and IL-4 were in the normal range in the first 5 weeks and started to fluctuate around the normal upper limit from week six (Fig. 2a, b). Fluctuations of TNF-α and IFN-γ in all the patients were minimal, and they were at very low levels from week 3 to week 7 (Fig. 2e, f). Elevation of IL-6 lasted for the whole observation course. In the severe group, it began to decline from week 6; in the critical group, it was sustained at a high level, especially in the nonsurvivors (Fig. 2c). A similar trend was seen in IL-10, and the nonsurvivors presented a significantly elevated level until death (Fig. 2d).

### Correlation of lymphocyte subsets and inflammatory cytokines

**Fig. 3:**
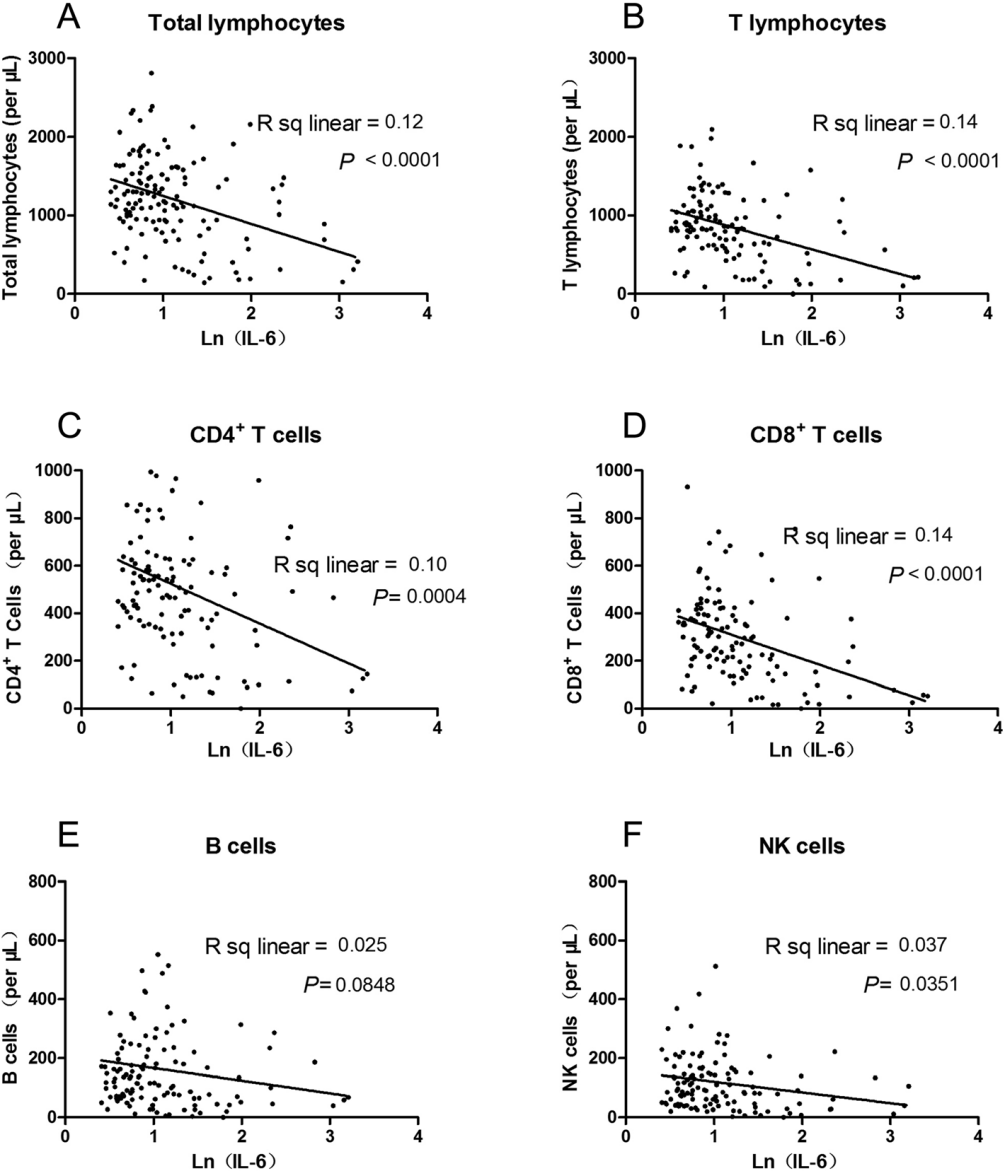
Correlation analysis between IL-6 and lymphocyte subsets. Correlation between Ln (IL-6) and total lymphocytes (**a**), T lymphocytes (**b**), CD4^+^ T-cells (**c**) and CD8^+^ T-cells (**d**), B cells (**e**), NK cells (**f**) were shown

**Fig. 4:**
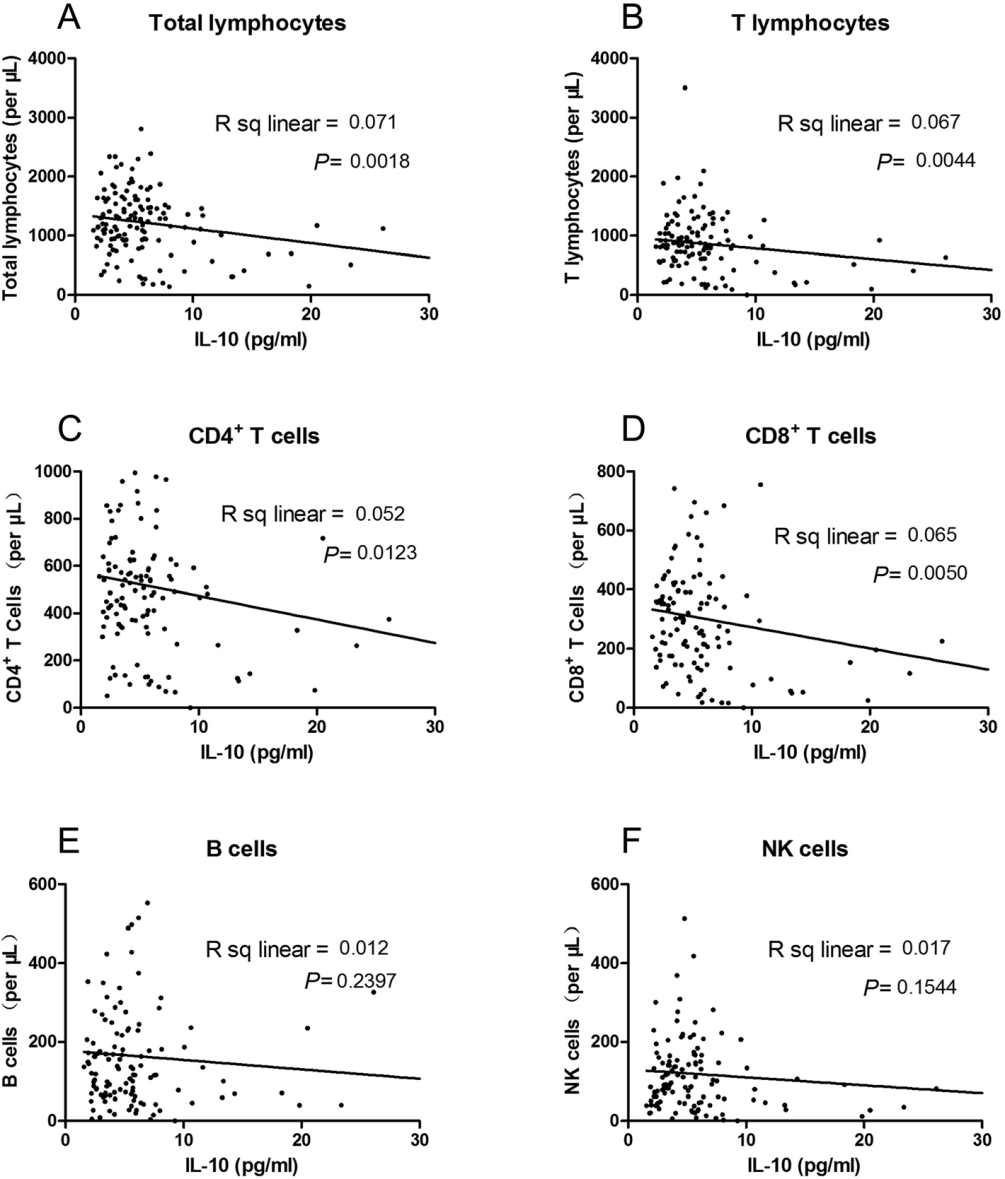
Correlation analysis between IL-10 and lymphocyte subsets. Correlation between IL-10 and total lymphocytes (**a**), T lymphocytes (**b**), CD4^+^ T-cells (**c**) and CD8^+^ T-cells (**d**), B cells (**e**), NK cells (**f**) were shown

To define possible associations among lymphocyte subsets and inflammatory cytokines, we assessed correlations between them. A strong negative correlation was observed between IL-6 and total lymphocytes, T lymphocytes, CD4^+^ T cells and CD8^+^ T cells. IL-6 was negatively correlated with NK cells as well, indicating that an inflammatory environment may suppress both innate (NK) and adaptive (CD3) immune cells (Fig. 3). Similar adverse effects were also observed between IL-10 and total lymphocytes, T lymphocytes, CD4^+^ T cells and CD8^+^ T cells (Fig. 4).

## Discussion

In this study, we longitudinally investigated the dynamics of peripheral immune cells and inflammatory cytokines in 67 COVID-19 patients with different disease severities and found that critical cases showed significantly decreased lymphocyte subsets and increased IL-6 and IL-10 levels on admission. Most of the parameters began to recover to comparable levels to those in the severe group from the fourth week after illness onset, except for NK cells, which recovered later from the sixth week. A sustained decrease in lymphocyte subsets and an increase in IL-6 and IL-10 were observed in the nonsurvivors until death. There was a strong negative correlation between IL-6, IL-10 and total lymphocytes, T lymphocytes, CD4^+^ T cells, CD8^+^ T cells and NK cells.

Lymphopenia was observed in most of the critical cases with COVID-19 on admission and could be a potential predictor for prognosis, as reported in recently published studies [8, 16–18]. Further analysis of the lymphocyte subsets also showed a significant decline in T lymphocytes, CD4^+^ T cells, CD8^+^ T cells, B cells, and NK cells associated with disease severity, indicating possible dysfunction of the immune response during SARS-CoV-2 infection. The mechanism of the reduction in lymphocytes might include immune injuries caused by deregulated cytokine production [1], up-regulation of apoptosis and autophagy in lymphocytes [19], lung tissue recruitment of immune cells [20] and destruction of lymphatic organs, including the spleen and lymph nodes [21]. Whether SARS-COV-2 can directly infect lymphocytes requires more detailed studies.

A recent meta-analysis demonstrated that alterations of lymphocyte subsets after treatment, rather than baseline levels, were reliable indicators for predicting COVID-19 progression or mortality [22]. In this study, by observing the dynamic changes in lymphocyte subsets during the course of COVID-19, we further confirmed the correlation between lymphocyte subset alterations and the severity of the disease. The lymphocyte subsets in the critical cases were always lower than those in the severe group. After treatment, they gradually increased to levels comparable to those in the severe group at the fourth week and then recovered to the normal level before discharge. However, the decrease was sustained in nonsurvivors, suggesting that the recovery of lymphocytes was associated with prognosis. Moreover, we observed a more significant and longer-lasting reduction in CD4^+^ T cells than in CD8^+^ T cells, suggesting that CD4^+^ T cells may be the primary participants in controlling SARS-CoV-2. This is in line with recent studies reporting that SARS-CoV-2–specific CD4^+^ T cell responses predominate over CD8^+^ T cell responses and that CD4^+^ T cell cytopenias are a characteristic change in severe COVID-19 [23, 24]. However, it is not yet conclusive which lymphocyte subsets are preferentially involved in either protective or pathogenic immunity. CD4^+^ and CD8^+^ T cell responses were both reported in the acute and early convalescent phases of SARS-COV-2 infection, dominated by a Th1 response [25]. A more profound understanding of T cell immunity would be beneficial for the design of vaccines and new treatment strategies. In addition, our observation of the dynamic changes of NK cells showed that NK cells were the last to return to normal, which might be partially explained by COVID-19 directly infecting NK cells and inhibiting their functions [26] due to the expression of angiotensin-converting enzyme 2 (ACE2) on NK cells [27]. The delayed recovery of NK cells may support the hypothesis that innate immunity plays a greater role in determining disease progression; therefore, intervention to promote the innate immune system should be further considered [28].

Cytokine release syndrome (CRS) is common in patients with severe COVID-19 and correlates with respiratory failure, ARDS, and adverse clinical outcomes [29]. Previous studies have reported that significantly higher inflammatory cytokines were associated with the disease severity of COVID-19 [30]. Our study also demonstrated that IL-6, IL-10 and CRP, a protein driven by IL-6, were all positively correlated with the severity of the disease. Moreover, while lymphocytes returned to the normal range 1 month after illness onset, the elevation of IL-6 and IL-10 in the critical group lasted for the whole observation course until clinical improvement and discharge. A sustained hyperinflammatory response in patients after discharge was also reported by Wen et al. [31]. Recently, a study from Italy reported that 10.9% (125/1146) of discharged patients had a persistent infection or recurrence of COVID-19; among them 23.2% developed new clinical symptoms [32]. Accordingly, the need for longitudinal observation of recovered patients was suggested to better understand the disease patterns. Additionally, we found that IL-2 and IL-4 were elevated and exceeded the normal upper limit at the same time point of recovery of lymphopenia before discharge, indicating that they might be beneficial to the recovery process. IFN-γ and TNF-α were well below the reference range during the whole observation period as previously described [33], suggesting an immunosuppressive state with a decreased capacity to clear the virus.

IL-6 and IL-10 may impact the protective immune response by reducing the number of NK and T cells, thereby reducing the cytotoxic activity against infected cells [34], supported by our analysis that IL-6 and IL-10 were negatively correlated with T lymphocyte subsets and NK cells. Indeed, IL-6 has been confirmed to block lymphopoiesis [35] and inhibit natural killer cell cytotoxicity in humans [36]. IL-10 was also shown to play a suppressive role in T cell proliferation [37]. Based on the evidence that hyperinflammatory responses can lead to a cytokine storm and subsequent exhaustion of immune cells, therapeutic approaches targeting cytokines have attracted substantial investment. Among them, the IL-6 antagonist tocilizumab has been the most widely explored. However, the findings of published randomized controlled trials (RCTs) [38–40] seem controversial. While Stone et al.’s study [38] showed no mortality benefit in moderately ill patients with COVID-19, Hermine et al. found that tocilizumab may reduce the 14-day but not 28-day mortality [39]. More evidence is needed to determine the patient populations who might benefit most from treatments with tocilizumab, and a deeper understanding of the immune responses of SARS-COV-2 infection would help provide this important insight.

This study had several limitations. First, the retrospective design has inherent limitations of information biases and possible confounding data. Second, since the median time from illness onset to admission was 14 days in our study, lymphocyte subset and cytokine data in the first 2 weeks were too limited to analyse. Third, although no effective treatment was proven, the effects of antiviral drugs and corticosteroids need to be further explored. Thus, well-designed prospective studies should be performed to better understand the pathogenesis of COVID-19.

## Conclusions

In conclusion, a sustained decrease in lymphocyte subsets, especially CD4^+^ T cells and NK cells, interacting with proinflammatory cytokine storms was associated with severe disease and poor prognosis in COVID-19. These findings might be helpful in elucidating the disease pathogenesis of COVID-19 and in developing new therapeutic strategies.

## Data Availability

The datasets used during the current study are available from the corresponding author on reasonable request.

## Abbreviations

COVID-19: Coronavirus disease 2019
SARS-CoV-2: Severe acute respiratory syndrome coronavirus
ARDS: Acute respiratory distress syndrome
MERS: Middle East respiratory syndrome
RT-PCR: Real-time reverse-transcriptase polymerase chain reaction
IL: Interleukin
TNF-α: Tumor necrosis factor α
IFN-γ: Interferon γ
ICU: Intensive care unit
CRS: Cytokine release syndrome
CRP: C-reactive protein
LLN: Lower limit of normal
ULN: Upper limit of normal
